# Autonomic nervous system-mediated effects of galanin-like peptide on lipid metabolism in liver and adipose tissue

**DOI:** 10.1038/srep21481

**Published:** 2016-02-19

**Authors:** Satoshi Hirako, Nobuhiro Wada, Haruaki Kageyama, Fumiko Takenoya, Yoshihiko Izumida, Hyounju Kim, Yuzuru Iizuka, Akiyo Matsumoto, Mai Okabe, Ai Kimura, Mamiko Suzuki, Satoru Yamanaka, Seiji Shioda

**Affiliations:** 1 Department of Health and Nutrition, University of Human Arts and Sciences, Saitama, Japan; 2 Department of Internal Medicine, Graduate School of Medicine, The University of Tokyo, Tokyo, Japan; 3 Faculty of Health Care, Kiryu University, Gunma, Japan; 4 Department of Exercise and Sports Physiology, Hoshi University School of Pharmacy and Pharmaceutical Science, Tokyo, Japan; 5 Department of Clinical Dietetics & Human Nutrition, Faculty of Pharmaceutical Sciences, Josai University, Saitama, Japan; 6 Tokyo Shokuryo Dietitian Academy, Tokyo, Japan; 7 Hoshi University School of Pharmacy and Pharmaceutical Sciences Global Research Center for Innovative Life Science Peptide Drug Innovation, Tokyo, Japan; 8 Department of Biochemistry, Showa University School of Medicine, Tokyo, Japan

## Abstract

Galanin-like peptide (GALP) is a neuropeptide involved in the regulation of feeding behavior and energy metabolism in mammals. While a weight loss effect of GALP has been reported, its effects on lipid metabolism have not been investigated. The aim of this study was to determine if GALP regulates lipid metabolism in liver and adipose tissue via an action on the sympathetic nervous system. The respiratory exchange ratio of mice administered GALP intracerebroventricularly was lower than that of saline-treated animals, and fatty acid oxidation-related gene mRNA levels were increased in the liver. Even though the respiratory exchange ratio was reduced by GALP, this change was not significant when mice were treated with the sympatholytic drug, guanethidine. Lipolysis-related gene mRNA levels were increased in the adipose tissue of GALP-treated mice compared with saline-treated animals. These results show that GALP stimulates fatty acid β-oxidation in liver and lipolysis in adipose tissue, and suggest that the anti-obesity effect of GALP may be due to anorexigenic actions and improvement of lipid metabolism in peripheral tissues via the sympathetic nervous system.

Obesity increases the risk of developing diabetes, fatty liver, hyperlipidemia, metabolic syndrome and atherosclerotic diseases^1^,^2^. Preventing obesity thus forms an important aspect of maintaining a healthy body. Atherosclerotic diseases such as ischemic heart disease and cerebral infarction are the most common causes of death in many countries, and it is well known that hyperlipidemia is closely associated with these diseases^3^. Energy constancy is not controlled by a single organ, but rather via the coordinated action of various organs. Energy regulation within peripheral tissues, such as the liver, white adipose tissue (WAT) and brown adipose tissue (BAT), is coordinated by sympathetic and vagal parasympathetic nerve activity. The sympathetic nervous system (SNS) modulates both glucose production and glucose uptake in peripheral tissues^4^,^5^,^6^,^7^, and enhances fatty acid oxidation in the liver, lipolysis in WAT, and thermogenesis in BAT^8^,^9^. When energy levels are insufficient, lipids stored in adipocytes are hydrolyzed and released into the blood in the form of non-esterified fatty acids (NEFA) and glycerol^10^,^11^. Recently, it was reported that a liver–brain–adipose neural axis exists, whereby the liver sends signals to the brain via the vagus nerve, and the brain sends signals via sympathetic nerves to adipose tissue^12^,^13^,^14^.

Galanin-like peptide (GALP), a 60-amino acid neuropeptide that was originally isolated and identified from porcine hypothalamic extracts, has been shown to affect food intake regulation, energy metabolism and reproduction^15^,^16^,^17^,^18^. Following the discovery of GALP, various studies on its physiological function reported that the intracerebroventricular (i.c.v.) injection of GALP in mice results in a decrease in food intake and body weight^19^,^20^,^21^,^22^. In rodents, fasting reduces GALP mRNA expression and the number of neurons expressing GALP^23^. Conversely, leptin administration increases the number of GALP-expressing neurons in the brains of fasted rats compared to control fasted rats that were injected with saline^23^, indicating that GALP expression is regulated by leptin. Furthermore, in *ob/ob* mice, body weight and food intake decreased continuously following chronic GALP administration for 14 days^24^. The i.c.v. administration of GALP also produces a decrease in body weight at 24 h, possibly due to its effects on thermogenesis^25^. Recently, we found that GALP influenced the respiratory exchange rate (RER). The RER is calculated by the ratio of CO_2_ produced to O_2_ consumed, and reflects main source of metabolic energy such as carbohydrate, protein and fat. That is, when pure carbohydrate is consumed as the energy source, RER is theoretically 1.0. In contrast, under conditions where fat is included as a source of energy, RER decreases from 1.0 to 0.72 as the proportion constituted by fat increases. In a previous study, we reported that the RER decreased following the i.c.v. administration of GALP in mice^26^. This finding suggests that lipid metabolism is also regulated by GALP. In *ob/ob* mice, chronic administration of GALP increases uncoupling protein (UCP)1 gene and protein expression in BAT^24^, indicating that the enhanced energy metabolism induced by GALP takes place via sympathetic activation. It is therefore possible that GALP acts on peripheral tissues via autonomic nervous system activity. Despite these findings, the effects of GALP on lipid metabolism in other organs have not been investigated. In this study, we show that GALP affects lipid metabolism in the liver and WAT via its action on the SNS.

## Results

### Effect of GALP on lipid metabolism in peripheral tissues

**Figure 1:**
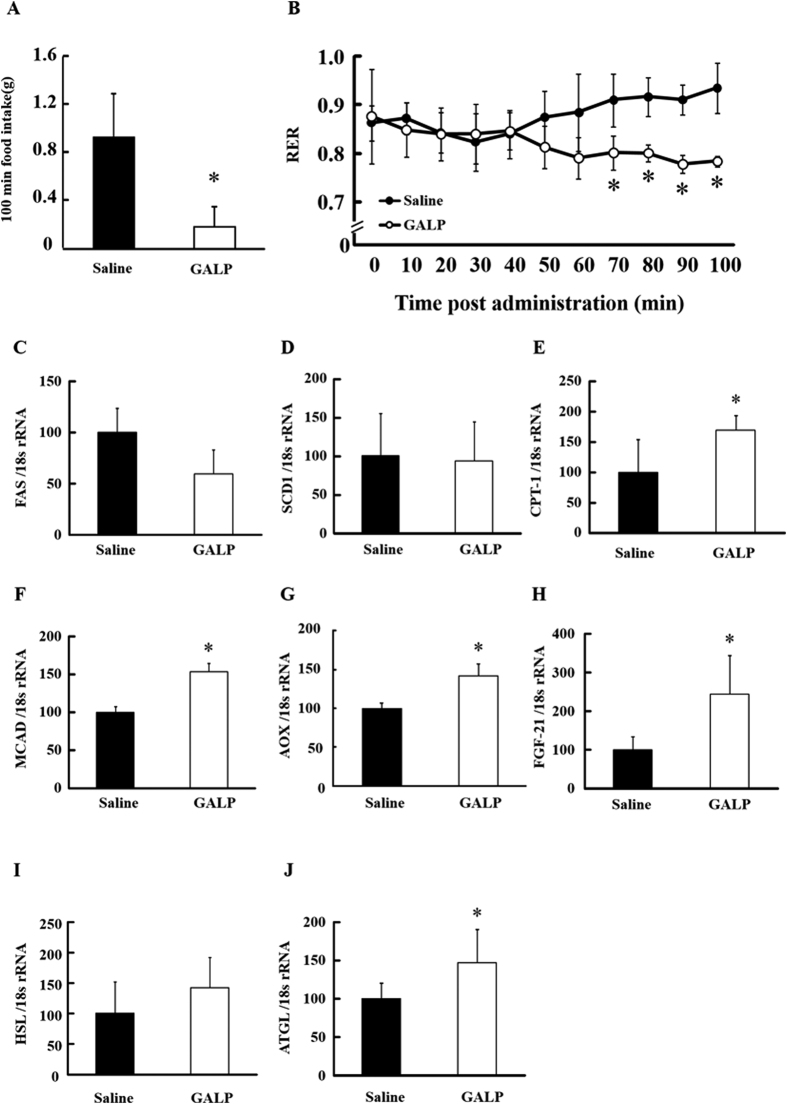
Effect of the i.c.v. administration of GALP on food intake, respiratory metabolism and mRNA expression levels in liver and WAT. Food intake (**A**) and respiratory exchange ratio (RER) (**B**) after i.c.v. administration of saline or 2 nmol of GALP. Expression levels of hepatic FAS (**C**), SCD1 (**D**), CPT-1 (**E**), MCAD (**F**), AOX (**G**), FGF-21 (**H**) genes and adipose HSL (**I**) and ATGL (**J**) genes were measured by real-time PCR, and expressed relative to 18S rRNA. Values represent means ± S.D. (n = 6). *p < 0.05 versus saline.

Energy and lipid metabolism were studied in mice administered a single i.c.v. dose of GALP or saline vehicle. One hundred minutes after the injection procedure to the brain, food intake was found to be reduced in the GALP-treated group with respect to the saline-treated control group (Fig. 1A). The RER of the GALP group was also lower than that of the saline group at around 60 min post-administration, and this decrease continued until animals were sacrificed (100 min post-i.c.v. injection) (Fig. 1B). This result suggested that fat is included as a source of energy by GALP treatment.

In relation to lipid components in the plasma, triglyceride (TG), total cholesterol (TC), NEFA and leptin levels were not significantly different between the GALP- and saline-treated groups, although there was a tendency for plasma fibroblast growth factor (FGF)-21 levels to increase in the GALP-treated group (*p* = 0.122; Table 1). Moreover, there was no significant difference between the groups in relation to hepatic TG and TC contents (Table 1). Expression levels of lipid metabolism-regulating genes in the liver and WAT are shown in Fig. 1C–J. The expression of mRNA for hepatic fatty acid synthase (FAS), a key enzyme in lipogenesis, was significantly down-regulated in the GALP-treated group compared with the saline group (Fig. 1C). However, no difference in expression levels of stearoyl-CoA desaturase (SCD)1 was observed between the saline- and GALP-treated groups (Fig. 1D). Levels of carnitine palmitoyltransferase (CPT)-1, medium-chain acyl-CoA dehydrogenase (MCAD) and acyl-CoA oxidase (AOX) mRNA, the proteins of all of which are involved in fatty acid oxidation, were significantly increased in the GALP-treated group compared with the saline group (Fig. 1E–G). FGF-21 mRNA expression was also significantly increased in the GALP-treated group (Fig. 1H). In adipose tissue, mRNA levels of hormone-sensitive lipase (HSL) and adipose triglyceride lipase (ATGL), which are involved in lipolysis, were increased in the GALP-treated group compared with control (Fig. 1I,J).

### Effect of GALP on lipid metabolism under short fast conditions

**Figure 2:**
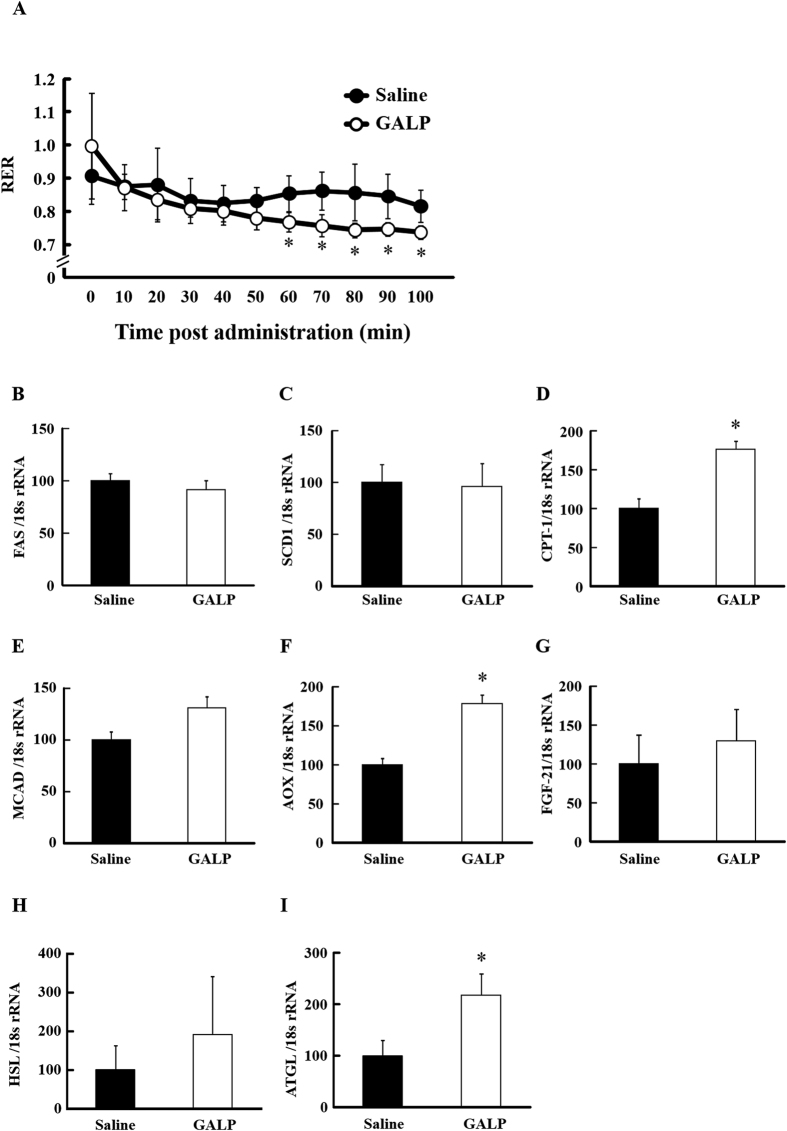
Effect of GALP i.c.v. administration on respiratory metabolism in short fast mice. Respiratory exchange ratio (RER) (**A**) after i.c.v. administration of saline or 2 nmol of GALP in short fast mice. Expression levels of hepatic FAS (**B**), SCD1 (**C**), CPT-1 (**D**), MCAD (**E**), AOX (**F**) and FGF-21 (**G**) and adipose HSL (**H**) and ATGL (**I**) were measured by real-time PCR, and expressed relative to 18S rRNA. Values represent means ± S.D. (n = 4). *p < 0.05 versus saline.

Based on the above results, we next examined the effects of GALP on lipid metabolism under short fast conditions. RER was significantly reduced in the GALP-treated group from approximately one hour after GALP administration compared to the saline-treated group (Fig. 2A). Hepatic FAS and SCD1 levels were not significantly different between the groups, whereas CPT-1 and AOX mRNA levels were significantly increased in the GALP group compared with the saline group, and MCAD and FGF-21 showed a trend to increase (Fig. 2B–G). mRNA levels of HSL and ATGL in adipose tissue were increased in the GALP-treated group compared with control (Fig. 2H,I). The expression of ATGL mRNA in particular was significantly increased.

### GALP-mediated effects on lipid metabolism in peripheral tissues via actions on the sympathetic nervous system

**Figure 3:**
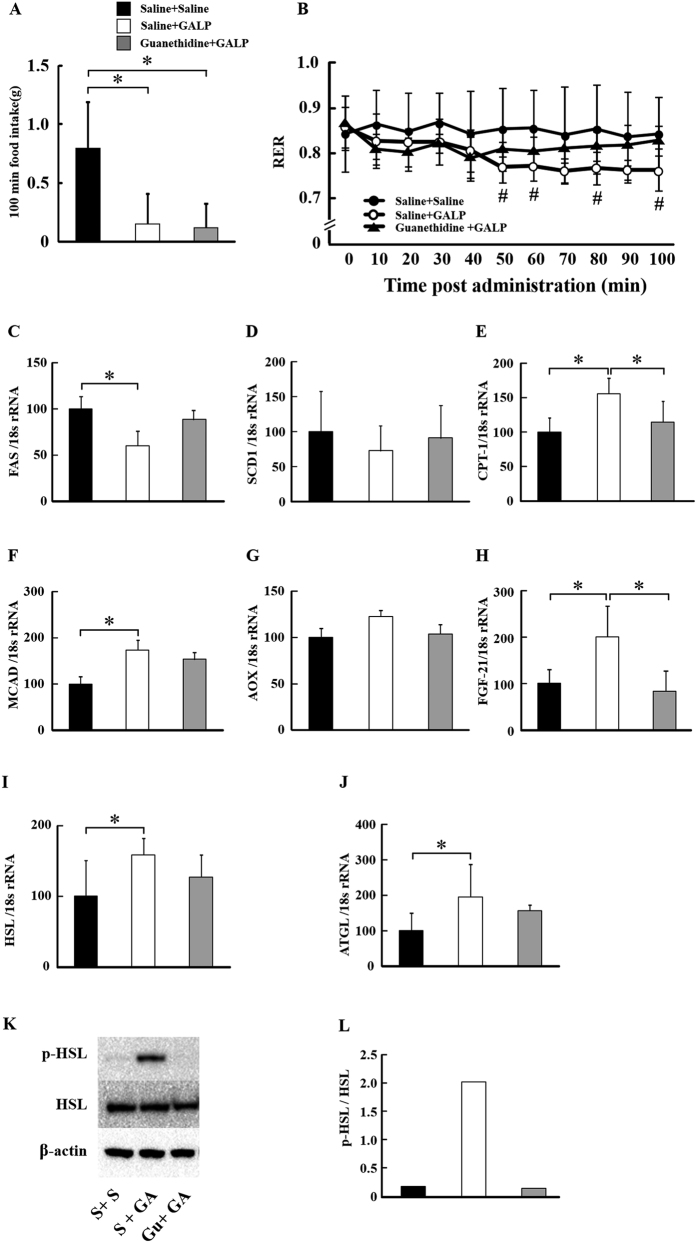
Effect of i.c.v. administration of GALP on food intake and respiratory metabolism in saline pretreatment and saline treatment group (Saline + Saline (S + S)), saline pretreatment and GALP treatment group (Saline + GALP (S + GA)) and guanethidine pretreatment and GALP treatment group (guanethidine + GALP (Gu + GA)). Food intake (**A**) and respiratory exchange ratio (RER) (**B**) after i.c.v. administration of saline or 2 nmol of GALP. Expression levels of hepatic FAS (**C**), SCD1 (**D**), CPT-1 (**E**), MCAD (**F**), AOX (**G**), FGF-21 (**H**) genes and adipose HSL (**I**) and ATGL (**J)** genes were measured by real-time PCR, and expressed relative to 18S rRNA. Expression of Ser 563 p-HSL and total HSL protein in epididymal white adipose tissue (**K,L**) (three individual protein samples pooled). Values represent means ± S.D. (n = 6). *P < 0.05; #, *P* < 0.05 versus the Saline + Saline group.

The adrenergic blocker guanethidine was used to examine the action of GALP on SNS-modulated lipid metabolism (Fig. 3). Food intake in the 100 minutes following i.c.v. administration was reduced in the GALP-treated group in the presence and absence of guanethidine (Fig. 3A). There was no significant difference between the Saline + Saline group and the Guanethidine + GALP group. RER was also reduced in this group compared to control in a manner that was suppressed by guanethidine treatment (Fig. 3B). Moreover, compared to control, FAS mRNA expression was reduced in the GALP-treated group, while SCD1 mRNA expression was unaffected, and CPT-1, MCAD and FGF-21 mRNA expression levels were increased (Fig. 3C–H). These actions of GALP could be inhibited by guanethidine treatment. HSL and ATGL mRNA levels were also increased in GALP-treated animals, and this effect was also suppressed by guanethidine (Fig. 3I,J). GALP i.c.v. administration dramatically increased the phosphorylation of HSL in WAT compared to that measured in saline-treated animals, and this too could be suppressed by guanethidine treatment (Fig. 3K,L).

## Discussion

This study shows that the i.c.v. administration of GALP stimulates hepatic fatty acid β- oxidation-related gene expression and lipolysis in WAT. Following the i.c.v administration of GALP, the RER began to decrease approximately one hour later and continued to decrease until animals were sacrificed 100 min post-administration. This result indicates that GALP was potentially involved in promoting fat consumption to provide energy. A decrease in RER with GALP administration was also observed under short fast conditions. Additionally, an increased expression of hepatic fatty acid β-oxidation-related genes (CPT-1, MCAD and AOX). These data suggest that GALP administered to the brain activates hepatic fatty acid β-oxidation, resulting in the consumption of fat for energy.

With regard to fatty acid synthesis, hepatic FAS and SCD1 mRNA expression levels were decreased in GALP-treated animals with *ad libitum* access to food. Previous studies have indicated that expression levels of genes related to fatty acid synthesis and β-oxidation vary with repeated fasting. In particular, hepatic fatty acid synthesis-related gene expression decreased during fasting^27^,^28^, whereas the expression of fatty acid β-oxidation-related genes increased under fasting conditions^27^,^29^,^30^,^31^. For this reason we examined the effect of GALP under short fast conditions, where food was removed after the i.c.v. administration of GALP or vehicle. Although the mouse enters a short-term fasted state at this time, we confirmed that GALP administration had no effect on lipid metabolism after a period of overnight fasting (results not shown). In this way, under the short fast condition, the decrease in fatty acid synthesis-related gene expression by GALP was not observed. Consequently, the decreased FAS and SCD1 gene expression observed in GALP-treated animals in our study may be due to a reduced food intake. GALP did however induce a decrease in RER and increased fatty acid β-oxidation-associated gene expression under short fast conditions. In other words, the major effect of GALP on lipid metabolism in the liver is increased fatty acid β-oxidation. Although hepatic β-oxidation-related gene expression was increased, plasma and liver lipid levels did not change significantly. The 100-min period between GALP administration and sacrifice of animals might have been too brief to enable changes in lipid concentrations to become clearly evident. In relation to WAT, expression levels of lipolysis-related genes and phosphorylated HSL protein were up-regulated in the GALP-treated group compared with control. On the other hand, plasma NEFA levels were not significantly different between the groups. We hypothesized that GALP would enhance lipolysis in adipose tissue and release fatty acids into the blood, which would then undergo fatty acid β-oxidation in the liver. It is thought that one of the anti-obesity effects of GALP could take place via this mechanism. However, it is possible that increased plasma NEFA levels were not observed in the present study because we only measured them for 100 minutes after GALP administration. Examination of the long-term effects of GALP on plasma NEFA and lipid levels will be an objective of future research.

The SNS has a clear association with lipid metabolism in the periphery^8^,^9^. It was reported that leptin activates hepatic 5′-adenosine monophosphate-activated protein kinase (AMPK) through α-adrenergic receptor-mediated effects on sympathetic nerves, promoting fatty acid β-oxidation in the liver^32^,^33^,^34^. In addition, SNS stimulation is known to enhance lipolysis in WAT^9^, with several reports attesting to such actions. For example, GALP administration increases body temperature and heart rate^35^. Also, the expression of UCP1 in BAT was increased by the i.c.v. administration of GALP^24^. These findings are consistent with sympathetic-mediated responses, implying that GALP can influence peripheral organs via actions on the SNS. To determine if this was the case in the present study, mice were treated with the peripheral sympathetic blocker, guanethidine. The reduction in RER observed in GALP-treated mice was suppressed by pre-treatment with guanethidine. On the other hand, the GALP-induced decrease in food intake was unaffected by guanethidine. It is thought that guanethidine inhibits the function of postganglionic adrenergic neurons, thus inhibiting peripheral sympathetic function and reversing the GALP-induced effects on RER. Food intake on the other hand, is centrally modulated by GALP and therefore remained unaffected by guanethidine. Our data also showed that guanethidine reversed the GALP-induced down-regulation of hepatic genes involved in fatty acid synthesis and up-regulated genes involved in fatty acid β-oxidation. Similarly, pre-treatment with guanethidine inhibited the GALP-induced phosphorylation of HSL in WAT. Taken together, these findings demonstrate that the actions of GALP on fatty acid β-oxidation in the liver and lipolysis in adipose tissue are mediated by sympathetic nerve stimulation.

Plasma FGF-21 levels showed a tendency to increase and hepatic FGF-21 mRNA expression was significantly increased by the GALP treatment. FGF-21 is synthesized in the liver and promotes lipolysis in adipose tissue; it is known that FGF-21 is a target gene of peroxisome proliferator-activated receptor (PPAR)α^36^,^37^,^29^. As expression levels of PPARα target genes such as CPT-1, AOX, MCAD, and FGF-21 were increased in GALP-treated animals, it is possible that GALP activated PPARα. These results suggest that GALP exerts it effects directly via the SNS and indirectly via the release of FGF-21 from the liver, which stimulates lipolysis in WAT. While it is known that leptin enhances fatty acid β-oxidation in the liver, it is suggested that the hepatic β-oxidation- related gene expression increasing effect of GALP was not mediated via an upregulation of leptin as no changes in plasma leptin levels were seen. The results shown here indicate that GALP acting within the brain controls lipid metabolism in peripheral tissues via the SNS. Nevertheless, the mechanism(s) by which this occurs remain(s) unknown. While the i.c.v. administration of GALP affects fatty acid β-oxidation in the liver and lipolysis in WAT via SNS-mediated actions, it should be noted that these tissues are also controlled by nerves that innervate the pneumogastric system. This, coupled with the fact that the actions of GALP were not fully inhibited by guanethidine, suggests that further studies are required to elucidate how GALP exerts its anti-obesity effects.

In conclusion, we have described here that GALP enhances fatty acid β-oxidation-related gene expression in the liver and lipolysis in the WAT. These newly observed physiological functions are exerted via actions on the SNS and via FGF-21-mediated humoral control, and may explain the previously reported anti-obesity effects of GALP. GALP may serve as a therapeutic option in the prevention and cure of obesity and dyslipidemia in clinical practice in the future.

## Methods

### Animals

Male C57BL/6J mice (5 or 9 weeks of age; Sankyo Labo Service Corporation, Inc., Tokyo, Japan) were used in all experiments. Mice were maintained on a 12-h day cycle (8:00 AM–8:00 PM) under conditions of 20 °C and 40–70% humidity, and allowed free access to standard diet (Labo MR stock; Nosan Corporation, Japan) and water. Following the i.c.v. administration of GALP or saline vehicle, food consumption was measured until sacrifice. In the short fast experiment, the energy intake of all mice was adjusted by removing the diet after i.c.v. administration. All mice were individually housed in metabolism cages allowing respiratory metabolism to be measured. All animal studies were conducted in accordance with the “Standards Relating to the Care and Management of Experimental Animals” (Notice No. 6 of the Office of Prime Minister dated March 27, 1980) and with approval from the Animal Use Committee of Showa University. (Approval Number: 04055).

### Intracerebroventricular (i.c.v.) injections

Mice were anesthetized with pentobarbital sodium and a small hole was bored in the skull 0.2 mm posterior and 1.1 mm laterally to the bregma for the positioning of an indwelling cannula (Laboratory and Medical Supplies Co., Ltd., Tokyo, Japan). Animals were allowed to recover for 7 d during which time they were handled daily. Placement of the guide cannula was checked by a positive drinking response to angiotensin II. Animals were then assigned randomly to a saline- (vehicle) or a GALP-treated group. After a washout period of 7 d, a single administration of 2 μL saline or GALP (1 nmol/μL), respectively, was given via the guide cannula at 18:30 h.

### Respiratory metabolism

Oxygen consumption (VO_2_) and carbon dioxide production (VCO_2_) were monitored using an indirect calorimeter (Oxymax, Columbus Instruments, Columbus, OH) in the 100 minutes following the saline or GALP administration. RER was calculated as the molar ratio of VCO_2_/VO_2_. In previous studies, the difference in RER value between the GALP and control groups reached a maximum about 100 minutes after administration^26^, for which reason mice in this study were sacrificed 100 minutes after the GALP or vehicle administration.

### Collection of blood and tissue samples

Blood samples were collected from the hearts of sacrificed animals and treated with heparin. The liver and epididymal WAT were removed, immediately frozen in liquid nitrogen, and stored at −80 °C until analysis. Plasma was obtained by centrifugation (900 × *g*, 4 °C, 10 min) and frozen at −80 °C.

### Quantification of liver and plasma lipids

A portion of liver tissue from each mouse was used for analyzing TG and total TC contents. Hepatic lipids were extracted from approximately 100 mg of liver tissue for each mouse in accordance with the method of Folch *et al*.^38^. Quantification of liver and plasma TG and TC were performed using the Triglyceride E-Test and Cholesterol E-Test kits, respectively, while plasma NEFA were quantified using the NEFA C-Test kit (Wako Pure Chemical Industries, Ltd). Plasma leptin levels were quantified by enzyme-linked immunosorbent assay (ELISA) using the Leptin/mouse ELISA kit (Morinaga Institute of Biological Science, Tokyo, Japan). FGF-21 levels in plasma were measured with the FGF-21 ELISA kit (R&D System, Minneapolis, USA).

### Quantification of mRNA expression

Total RNA was extracted from liver and WAT tissue from each mouse using Trizol (Life Technologies, Inc.) in accordance with the manufacturer’s protocol, and then converted into cDNA with an Affinity Script QPCR cDNA Synthesis Kit (Stratagene, Agilent Technologies, La Jolla).

Quantification of mRNA expression levels by TaqMan real-time PCR was performed using an ABI7900HT instrument (PE Applied Biosystems, Foster City, CA). mRNA was amplified using EagleTaq Master Mix PCR reagent (Roche Applied Science, Indianapolis, IN). Primers are listed in Table 2. Universal ProbeLibrary probes were purchased from Roche Diagnostics GmbH. Target genes were normalised to the endogenous control (18s rRNA). The 18S rRNA primer and the VIC/MGB probe set were purchased from Life Technologies, Inc.

### Sympathetic nerve blockade

C57BL/6J mice received an intraperitoneal (i.p.) injection of guanethidine hemisulfate (Wako Pure Chemical Industries, Ltd) to block sympathetic nerve activity. Mice were pretreated with saline (vehicle) or saline containing (40 mg/ kg) guanethidine. Fifteen minutes later mice were given an i.c.v. injection of saline or GALP (2 nmol).

### Western blot

Western blotting was performed as reported previously^39^. Briefly, whole-cell lysates or tissue extracts were fractionated by SDS–PAGE and transferred to a polyvinylidene difluoride membrane using a transfer apparatus according to the manufacturer’s protocol (Bio-Rad). After blocking with 5% skim-milk in 1 × TTBS (tris-tween-buffer-saline) (10 × TTBS: NaCl, 80 g; 1 M Tris–HCl, pH 7.5, 200 mL; Tween-20, 5 mL) for 1 h, the membrane was incubated with primary antibody at 4 °C for 12 h. Membranes were washed five times for 10 min and incubated with a 1:10,000 dilution of horseradish peroxidase-conjugated second antibody for 1 h. For blot development, the luminol/enhancer and peroxide buffer solutions were mixed in a 1:1 ratio (1 mL:1 mL; one membrane volume) and spread over the membrane and incubated at RT for 5 min. Signals (cross-reacting protein bands) were visualized using the ChemiDoc XRS + imaging system (Bio-Rad, Hercules, CA, USA).

### Statistical Analysis

Differences between two groups were assessed using the unpaired two-tailed Student’s t-test unless otherwise noted. Data sets involving more than three groups (sympathetic nerve blockade study) were assessed by Tukey’s post-hoc test. Values were reported as the mean ± SD. Statistical significance was defined as *P* < 0.05.

## Additional Information

**How to cite this article**: Hirako, S. *et al*. Autonomic nervous system-mediated effects of galanin-like peptide on lipid metabolism in liver and adipose tissue. *Sci. Rep*. **6**, 21481; doi: 10.1038/srep21481 (2016).

## Figures and Tables

**Table 1:** Plasma parameters and liver lipid levels of mice administered i.c.v. with saline or GALP.

	Saline	GALP
Plasma		
Total cholesterol (mg/mL)	170.09 ± 19.7	171.79 ± 16.4
Triglyceride (mg/mL)	46.22 ± 13.77	49.72 ± 16.63
Non-esterified fatty acids (mEq/mL)	0.93 ± 0.25	1.13 ± 0.22
Leptin (ng/mL)	1.37 ± 0.66	1.30 ± 0.52
FGF-21 (pg/mL)	68.23 ± 39.17	110.94 ± 48.09
Liver		
Total cholesterol (mg/g)	2.86 ± 0.5	3.38 ± 0.49
Triglyceride (mg/g)	2.19 ± 1.29	2.67 ± 1.68

Values represent means ± S.D. (n = 6).

**Table 2:** Primers for RT-PCR amplification of indicated genes.

Gene	Forward primer(5′-3′)	Reverse primer(5′-3′)
FAS	gctgctgttggaagtcagc	agtgttcgttcctcggagtg
SCD1	ttccctcctgcaagctctac	cagagcgctggtcatgtagt
CPT-1	ggacattatcaccttgtttggc	ggagcaacacctattcatttgg
MCAD	agtaccctgtggagaagctgat	tcaatgtgctcacgagctatg
AOX	caccattgccattcgataca	tgcgtctgaaaatccaaaatc
FGF-21	agatggagctctctatggatcg	gggcttcagactggtacacat
HSL	gcgctggaggagtgttttt	cgctctccagttgaaccaag
ATGL	tgaccatctgccttccaga	tgtaggtggcgcaagaca

FAS: fatty acid synthase, SCD: stearoyl-CoA desaturase, CPT: carnitine palmitoyltransferase, MCAD: medium-chain acyl-CoA dehydrogenase, AOX: acyl-CoA oxidase, FGF-21: fibroblast growth factor-21, HSL: hormone-sensitive lipase, ATGL: adipose triglyceride lipase.

## References

[b1] Wilson P. W., D’Agostino R. B., Sullivan L., Parise H. & Kannel W. B. Overweight and obesity as determinants of cardiovascular risk: the Framingham experience. Arch. Intern. Med. 162, 1867–1872 (2002). 12196085 10.1001/archinte.162.16.1867

[b2] Meshkani R. & Adeli K. Hepatic insulin resistance, metabolic syndrome and cardiovascular disease. Clin. Biochem. 42, 1331–1346 (2009). 19501581 10.1016/j.clinbiochem.2009.05.018

[b3] Kodama K., Sasaki H. & Shimizu Y. Trend of coronary heart disease and its relationship to risk factors in a Japanese population: a 26-year follow-up, Hiroshima/Nagasaki study. Jpn Circ.J. 54, 414–421 (1990). 2398621 10.1253/jcj.54.414

[b4] Nonogaki K. New insights into sympathetic regulation of glucose and fat metabolism. Diabetologia 43, 533–549 (2000). 10855527 10.1007/s001250051341

[b5] Shimazu T. Innervation of the liver and glucoregulation: roles of the hypothalamus and autonomic nerves. Nutrition 12, 65–66 (1996). 8838845 10.1016/0899-9007(96)00060-3

[b6] Perseghin G. . Regulation of glucose homeostasis in humans with denervated livers. J. Clin. Invest. 100, 931–941 (1997). 9259593 10.1172/JCI119609 PMC508266

[b7] Nonogaki K. & Iguchi A. Role of central neural mechanisms in the regulation of hepatic glucose metabolism. Life Sci. 60, 797–807 (1997). 9076318 10.1016/s0024-3205(96)00596-6

[b8] Bartness T. J. & Bamshad M. Innervation of mammalian white adipose tissue: implications for the regulation of total body fat. Am.J. Physiol. 275, R1399–1411 (1998). 9791054 10.1152/ajpregu.1998.275.5.R1399

[b9] Lafontan M. & Berlan M. Fat cell alpha 2-adrenoceptors: the regulation of fat cell function and lipolysis. Endocr. Rev. 16, 716–738 (1995). 8747832 10.1210/edrv-16-6-716

[b10] Lafontan M. & Langin D. Lipolysis and lipid mobilization in human adipose tissue. Prog. Lipid. Res. 48, 275–297 (2009). 19464318 10.1016/j.plipres.2009.05.001

[b11] Ahmadian M., Wang Y. & Sul H. S. Lipolysis in adipocytes. Int. J. Biochem. Cell. Biol. 42, 555–559 (2010). 20025992 10.1016/j.biocel.2009.12.009 PMC2835819

[b12] Izumida Y. . Glycogen shortage during fasting triggers liver-brain-adipose neurocircuitry to facilitate fat utilization. Nat. Commun. 4, 2316 (2013). 23939267 10.1038/ncomms3316 PMC3753545

[b13] Uno K. . Neuronal pathway from the liver modulates energy expenditure and systemic insulin sensitivity. Science 312, 1656–1659 (2006). 16778057 10.1126/science.1126010

[b14] Tsukita S. . Hepatic glucokinase modulates obesity predisposition by regulating BAT thermogenesis via neural signals. Cell. Metab. 16, 825–832 (2012). 23217261 10.1016/j.cmet.2012.11.006

[b15] Ohtaki T. . Isolation and cDNA cloning of a novel galanin-like peptide (GALP) from porcine hypothalamus. J. Biol. Chem. 274, 37041–37045 (1999). 10601261 10.1074/jbc.274.52.37041

[b16] Takenoya F. . Galanin-like peptide is target for regulation by orexin in the rat hypothalamus. Neurosci. Lett. 340, 209–212 (2003). 12672543 10.1016/s0304-3940(03)00120-4

[b17] Takenoya F. . Galanin-like peptide is co-localized with alpha-melanocyte stimulating hormone but not with neuropeptide Y in the rat brain. Neurosci. Lett. 331, 119–122 (2002). 12361855 10.1016/s0304-3940(02)00867-4

[b18] Takenoya F. . Neuronal interactions between galanin-like-peptide- and orexin- or melanin-concentrating hormone-containing neurons. Regul. Pept. 126, 79–83 (2005). 15620418 10.1016/j.regpep.2004.10.004

[b19] Man P. S. & Lawrence C. B. The effects of galanin-like peptide on energy balance, body temperature and brain activity in the mouse and rat are independent of the GALR2/3 receptor. J. Neuroendocrinol. 20, 128–137 (2008). 18081561 10.1111/j.1365-2826.2007.01625.x PMC3306895

[b20] Krasnow S. M. . A role for galanin-like peptide in the integration of feeding, body weight regulation, and reproduction in the mouse. Endocrinology 144, 813–822 (2003). 12586757 10.1210/en.2002-220982

[b21] Krasnow S. M. . Analysis of the contribution of galanin receptors 1 and 2 to the central actions of galanin-like peptide. Neuroendocrinology 79, 268–277 (2004). 15249737 10.1159/000079632

[b22] Kauffman A. S., Buenzle J., Fraley G. S. & Rissman E. F. Effects of galanin-like peptide (GALP) on locomotion, reproduction, and body weight in female and male mice. Horm. Behav. 48, 141–151 (2005). 16042964 10.1016/j.yhbeh.2005.01.010

[b23] Takatsu Y. . Distribution of galanin-like peptide in the rat brain. Endocrinology 142, 1626–1634 (2001). 11250944 10.1210/endo.142.4.8089

[b24] Hansen K. R. . Activation of the sympathetic nervous system by galanin-like peptide–a possible link between leptin and metabolism. Endocrinology 144, 4709–4717 (2003). 12960003 10.1210/en.2003-0748

[b25] Lawrence C. B., Baudoin F. M. & Luckman S. M. Centrally administered galanin-like peptide modifies food intake in the rat: a comparison with galanin. J. Neuroendocrinol. 14, 853–860 (2002). 12421338 10.1046/j.1365-2826.2002.00846.x

[b26] Ito K. . Interactive effect of galanin-like peptide (GALP) and spontaneous exercise on energy metabolism. Peptides 49, 109–116 (2013). 24055807 10.1016/j.peptides.2013.09.003

[b27] Palou M. . Sequential changes in the expression of genes involved in lipid metabolism in adipose tissue and liver in response to fasting. Pflugers. Arch. 456, 825–836 (2008). 18493788 10.1007/s00424-008-0461-1

[b28] Ikeda I. . Impact of fasting time on hepatic lipid metabolism in nutritional animal studies. Biosci. Biotechnol. Biochem. 78, 1584–1591 (2014). 25209508 10.1080/09168451.2014.923297

[b29] Wang Y. X. PPARs: diverse regulators in energy metabolism and metabolic diseases. Cell Res. 20, 124–137 (2010). 20101262 10.1038/cr.2010.13 PMC4084607

[b30] Kersten S. . Peroxisome proliferator-activated receptor alpha mediates the adaptive response to fasting. J. Clin. Invest. 103, 1489–1498 (1999). 10359558 10.1172/JCI6223 PMC408372

[b31] Leone T. C., Weinheimer C. J. & Kelly D. P. A critical role for the peroxisome proliferator-activated receptor alpha (PPARalpha) in the cellular fasting response: the PPARalpha-null mouse as a model of fatty acid oxidation disorders. Proc. Natl. Acad. Sci. USA. 96, 7473–7478 (1999). 10377439 10.1073/pnas.96.13.7473 PMC22110

[b32] Miyamoto L. . Leptin activates hepatic 5′-AMP-activated protein kinase through sympathetic nervous system and alpha1-adrenergic receptor: a potential mechanism for improvement of fatty liver in lipodystrophy by leptin. J. Biol. Chem. 287, 40441–40447 (2012). 23024365 10.1074/jbc.M112.384545 PMC3504759

[b33] Tanida M., Yamamoto N., Shibamoto T. & Rahmouni K. Involvement of hypothalamic AMP-activated protein kinase in leptin-induced sympathetic nerve activation. PloS one 8, e56660 (2013). 23418591 10.1371/journal.pone.0056660 PMC3572050

[b34] Minokoshi Y. . Leptin stimulates fatty-acid oxidation by activating AMP-activated protein kinase. Nature 415, 339–343 (2002). 11797013 10.1038/415339a

[b35] Kageyama H. . Galanin-like peptide (GALP) facilitates thermogenesis via synthesis of prostaglandin E2 by astrocytes in the periventricular zone of the third ventricle. J. Mol. Neurosci. 50, 443–452 (2013). 23354880 10.1007/s12031-013-9952-4

[b36] Potthoff M. J. . FGF21 induces PGC-1alpha and regulates carbohydrate and fatty acid metabolism during the adaptive starvation response. Proc. Natl. Acad. Sci. USA. 106, 10853–10858 (2009). 19541642 10.1073/pnas.0904187106 PMC2705613

[b37] Lundasen T. . PPARalpha is a key regulator of hepatic FGF21. Biochem. Biophys. Res. Commun. 360, 437–440 (2007). 17601491 10.1016/j.bbrc.2007.06.068

[b38] Folch J., Lees M. & Sloane Stanley G. H. A simple method for the isolation and purification of total lipides from animal tissues. J. Biol. Chem. 226, 497–509 (1957). 13428781

[b39] Hirano M. . New protein extraction/solubilization protocol for gel-based proteomics of rat (female) whole brain and brain regions. Mol. Cells. 22, 119–125 (2006). 16951559

